# Manual of Clinical Diagnosis

**Published:** 1887-11

**Authors:** 


**Manual of Clinical Diagnosis.** By Dr. Otto Seifert and Dr. F. Mueller. Third edition, revised and corrected. Translated, with the permission of the author, by Wm. B. Canfield, A. M., M. D., Lecturer on Histology, etc., University, Maryland, with sixty illustrations. New York and London: G. P. Putnam's Sons. 1887.

This valuable little work gives, in an epitomized form, the different methods of clinical examination, as well as a convenient collection of those data and figures which are most valuable in every-day work. It is a book admirably adapted to the practical need of the physician and student, and we would most heartily commend it.

